# Reducing Employee Turnover Intentions in Tourism and Hospitality Sector: The Mediating Effect of Quality of Work Life and Intrinsic Motivation

**DOI:** 10.3390/ijerph191811222

**Published:** 2022-09-07

**Authors:** Ying Xu, Diao Jie, Hongyu Wu, Xiaolu Shi, Daniel Badulescu, Sher Akbar, Alina Badulescu

**Affiliations:** 1 Zhengzhou Preschool Education College, Zhengzhou 450002, China; 2 Yangxin Jingtoushan Farm School, Huangshi 435200, China; 3 Department of Economics and Business, Faculty of Economic Sciences, University of Oradea, 410087 Oradea, Romania; 4 Department of Management Sciences, COMSATS University Islamabad, Islamabad 45550, Pakistan

**Keywords:** mental health, CSR, turnover, stress, tourism and hospitality

## Abstract

Employee turnover causes various organizational disruptions, including economic and social loss and a deficit in organizational knowledge-skill inventory. Considering different forms of organizational disruptions associated with employee turnover, the contemporary literature on organizational sciences has shown serious concern in dealing with the challenge of employee turnover. However, shockingly, the employee turnover rate in the tourism and hospitality sector has been reported to be critically high even at a global level. Moreover, considering the customer-facing nature of this industry, employee turnover has more consequences for the tourism and hospitality sector compared to other segments of the economy. Past literature has acknowledged the role of employee-related corporate social responsibility (ERCSR) activities of an organization in influencing employee behavior. However, a critical knowledge gap in this domain still exists. That is, most of the prior studies tested the impact of ERCSR on positive employee behavior and did not test how ERCSR engagement in an organization may reduce employee turnover intentions, especially in a hospitality context. To fill this knowledge gap, this study aimed to investigate the relationship between ERCSR and employee turnover intentions in a hospitality sector of a developing country. Additionally, the mediating roles of quality of work life and intrinsic motivation were also tested in the above-proposed relationship. The hotel employees were the respondents in this survey who provided their responses related to the study variables on a self-administered questionnaire (*n* = 278). A hypothetical model was developed and analyzed with the help of the structural equation modeling technique. The results confirmed that ERCSR orientation of a hotel organization significantly reduces the turnover intentions of employees, whereas both quality of work life and intrinsic motivation buffered this association by producing mediating effects. These findings have different theoretical and practical implications, among which the most important implication is to realize the key role of ERCSR in reducing employees’ turnover intentions in a hospitality context. Various other implications are discussed in detail.

## 1. Introduction

The phenomenon of employee turnover has long existed in organizational science literature. However, in the recent past, the organizational interest in reducing employee turnover intentions has been mounting. Employee turnover intentions refer to the state of an employee in which he or she shows his or her willingness to quit a job or an organization [1]. Past research indicates that turnover has different negative consequences for an organization, including, but not limited to, reduced employee performance [2], and organizational performance [3]. Indeed, turnover in any form has been associated with rising economic cost and organizational disruption. Data on turnover indicate that the economic cost of employee turnover is huge, which varies between 90 to 200 percent of the salary of current employees in an organization [4]. Holtom and Burch [5] mentioned that with a mounting rate of employee turnover, the social fabric of an organization is lost. Moreover, organizations with high employee turnover lose the valuable knowledge asset that an existing employee has. Not only an organization loses intangible knowledge and skills when an employee leaves an organization, but also it undermines the operational efficiency of an organization [6]. These are few reasons due to which modern enterprises from every segment show serious concern in mitigating employee turnover.

When associated with the hospitality sector, perhaps turnover is one of the biggest evils that exist in this sector. Indeed, the hospitality sector is known for its out-sized employee turnover rate worldwide [7,8]. Besides the economic cost of turnover in the hospitality sector, a high employee turnover makes it very difficult for hotel management to satisfy their consumers by providing them with continuous service quality [9].

Although the above discussion on the negative consequences of a high employee turnover rate presents a difficult situation for the management in a hospitality context, current evidence also suggests that around 75% of the reasons why employees leave an organization could be prevented [10]. Wildes and Parks [11] indicated that employee turnover in an organization may significantly be reduced as an outcome of concerted efforts from the employer to improve the working environment to facilitate the employees. 

Prior literature suggests that in an organizational milieu, different organizational factors can significantly reduce the turnover intentions of employees [12,13]. In this respect, recent literature highlights the seminal role of employee-related corporate social responsibility (ERCSR) initiatives of an organization to influence their behavior [14,15]. 

Literature under this stream mainly highlights the benefits of ERCSR to influence the positive behavior of employees [16,17,18,19]. However, studies on the relationship between ERCSR and employee turnover intentions are sparse, especially in a hospitality context. In spite of some recent exceptions existing in the literature [20], this insufficient explanation is still limited in advancing the debate and reaching a consensus. To bridge this knowledge gap, the major objective of this study was to investigate the association between ERCSR and employee turnover intentions.

The role of quality of work life in influencing different employee outcomes was also highlighted in the available literature on organizational psychology [21,22]. Specifically, the literature on positive employee psychology indicates that employees’ positive perceptions about an improved quality of work life can enhance their mental health, reducing undesired work behaviors including burnout [23] and turnover intentions of employees [24]. 

Literature also emphasizes the importance of different psychological factors as mediators and moderators to understand specific individual behavior in a certain context. In this respect, although the mediating role of quality of work of life was recognized by early organizational scientists [25,26], nevertheless, such mediating effect in an ERCSR and turnover intentions framework, from the perspective of the hospitality sector, was not previously highlighted. Hence, another important objective of this study was to fill this knowledge gap.

Likewise, a growing body of literature related to employee wellbeing and mental health suggests that personality characteristics, like intrinsic motivation, can influence the employees’ capability to cope with different work-related stressors [27] that ultimately lead employees to quit a job or organization. Although the mediating effect of intrinsic motivation was established in prior literature [28,29], the mediating role of intrinsic motivation to reduce employee turnover, in a CSR framework, was not emphasized previously, indicating an important knowledge gap in the existing literature. The current study intends to fill this knowledge gap with an objective to investigate the mediating effect of intrinsic motivation between the relationship between ERCSR and employee turnover intentions in the hospitality sector. 

The hospitality sector of Pakistan has been selected to test the proposed relationships. This sector was selected based on the following specific reasons. First, like in other regions of the world, the turnover rate of employees in the country was reported to be higher compared to other service segments [30]. Thus, it will be worthwhile to reduce employee turnover intentions in this sector in a CSR framework. Second, from the perspective of the services industry, the hospitality sector is a consumer-facing sector where employees constantly maintain contact with different consumers. From this perspective, when consumers see that the staff of a hotel is constantly moving, it leaves a bad impression on them on one hand. It also makes it challenging for the management to serve the consumers with an unchanged service delivery pattern on the other hand, because in a service milieu, the quality of service delivery is more dependent on employees compared to a non-service context [31,32]. Hence, retaining employees in this sector is a serious matter of concern. Last, from an economic perspective, the cost of employee turnover is huge for an organization, therefore, an improvement in the employee turnover rate will ultimately provide an economic benefit to an organization. 

## 2. Theoretical Framework and Hypotheses 

A growing body of knowledge has employed the self-concept theory to anatomize the undesired work behavior of employees, including their turnover intentions. Specifically, organizational scientists have attempted to explain employee turnover intentions from the perspective of business or organizational ethics [33,34]. Rosenberg [35] first introduced this theory and argued that the self-concept of an individual is the totality of feelings of an individual and how he or she perceives himself or herself. From an organizational perspective, Shamir, et al. [36] related self-concept theory in an organizational milieu. According to them, the perceptions of an employee about others (an organization in the current context), also influence his or her self-concept. In this respect, an ethical organization under its CSR strategy works in the larger interest of society, the community, and all other stakeholders, including the employees. Based on the theory of self-concept, employee turnover intentions are influenced by the ethical context of an organization. In other words, the CSR philosophy of an ethical organization (the self-concept of an employee for an organization) influences their self-evaluation criterion positively [37]. Indeed, Lee and Lee [38] mentioned that an organization’s ethical conduct makes it possible for the employees to relate their self-concept with organizational goals and values. From this perspective, this congruence between employees’ self-concept and organizational goals reduces a value conflict between employees and an organization, ultimately reducing their intentions to quit their job [39]. Based on the above discussion, we feel this theory provides a theoretical underpinning to understand employee–employer relationships in an ethical context. 

Current evidence suggests that the CSR activities of an organization can influence different employee outcomes. Specifically, it was emphasized in the literature that employee-focused CSR (also known as micro-CSR) activities of an enterprise can significantly influence their work behavior [40,41]. Scholars under this micro stream of CSR have already indicated that ERCSR engagement of an organization could induce their pro-environmental behavior [7,42,43], creativity [44], innovative behavior [45], advocacy behavior [18], organizational citizenship behavior [46], and several other behaviors. Despite the fact that the literature on positive organizational psychology mentions different benefits of ERCSR, recently, it was realized by some behavioral scientists that ERCSR activities of an enterprise can help in reducing the undesired work-related outcomes on the part of employees. For example, it was mentioned in the available CSR literature that employees in an ethical organization feel less stress [47], violence [48], burnout [49,50], etc. In a similar vein, the literature suggests that ERCSR can help an organization to reduce employee turnover intentions [51,52].

Accordingly, an organization, under its ERCSR policy, provides its employees with different benefits, including a flexible, healthy, and safe working environment, employee promotion plan, training and development, talent development, etc. [53]. Not only does an organization provide these benefits to the employees under its ERCSR policy, but the ethical context of an organization also improves the employees’ mental health. Being associated with an ethical organization, employees feel entrusted that they will be treated fairly without any prejudice. Further, employees believe that in a socially responsible organization, they will be treated with respect and care [54]. They also believe that their organization shows a concern for the betterment of its internal stakeholders [40]. In one way or another, all these factors enhance employees’ mental health. Literature indicates that employees with an improved level of mental health are less likely to think about leaving their job [55,56]. Therefore, it can be stated:

**H1.**  *ERCSR activities of an organization reduce employee turnover intentions.*

Additionally, an organization that takes into consideration the welfare aspect of its employees is expected to raise employees’ perceptions about the quality of work life [57]. Thus, such organizations foster the motivation and commitment level of their employees through different employee benefit programs under ERCSR policies. Kara, et al. [58] referred to the quality of work life as the extent to which employees feel freedom and flexibility to perform their job tasks in relation to personal needs and interests. According to Sanda and Majoreen [59], an organization’s quality of work-life program for its employees intends to enhance their satisfaction and mental health by providing a suitable working environment. The literature identifies different organizational factors like organizational policies, procedures, leadership style, operations, and others that affect employees’ perception of their quality of work-life in an organization [60]. At the same time, it was mentioned in the recent literature that employees’ perceptions of quality of work life significantly improve as an antecedent of a firm’s CSR strategies [61,62]. Specifically, Kim et al. [63] mentioned that in a hospitality context, employees positively evaluate the CSR activities of a hotel organization which then improves their quality of work-life perceptions about a socially responsible hotel. The authors like Lee, Sirgy and Senasu [5] also presented the same kind of arguments. Literature also indicates that employees with an improved perception of quality of work life with an organization are expected to stay with such an organization as long as possible [64]. Even from a hospitality perspective, Kim, et al. [65] mentioned that quality of work life not only directly affects employees’ intentions to stay in an organization but also mediates between CSR and employees’ intentions to stay with a hotel organization. The other scholars in the domain of employee wellbeing also indicated the mediating role of quality of work life in a CSR framework [66]. All in all, as the mediating effect of quality of work life has an established link in prior literature on CSR, employees with an improved quality of work life perceptions are less likely to quit an organization, therefore, we propose:

**H2.**  *ERCSR policies of an organization improve employees’ quality of work-life perceptions.*

**H3.**  *Quality of work-life perceptions of employees mediate between ERCSR and turnover intentions.*

**Figure 1:**
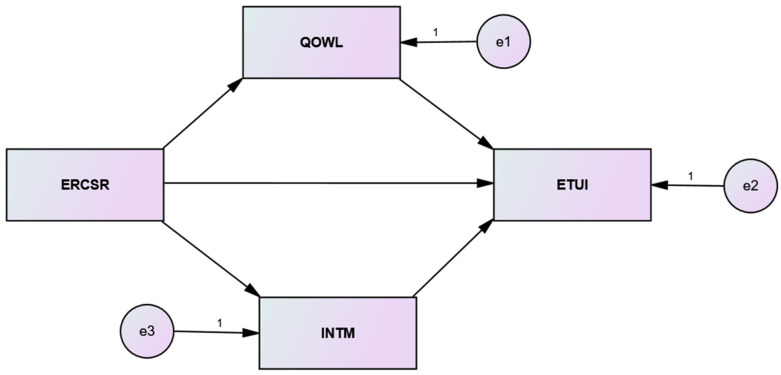
The hypothesized structural model: ERCSR = Ethical leadership (X); QOWL = Quality of work life (M1); INTM = Intrinsic motivation (M2); ETUI = Employee turnover intentions (Y).

Literature on employee motivation generally discusses two motivational aspects: extrinsic motivation and intrinsic motivation [67,68]. Whereas the extrinsic motivation of employees relates to external rewards, for example, salary and promotions, the inward feelings of respect, pride, and self-commitment are the subjects of intrinsic motivation. Deci and Ryan [69] provided an academic definition of intrinsic motivation (which is emphasized in this study). According to them, it is a process in which employees are internally motivated to complete a task. They further asserted that an intrinsically motivated employee shows extra commitment to complete a task for his or her inner satisfaction, not for the external rewards. In an organizational milieu, it was mentioned in the past literature that the level of employees’ intrinsic motivation improves as their CSR perceptions of their organization improve [70,71]. Especially the literature on positive employee psychology indicates that employees positively evaluate their organization’s CSR engagement and feel pride in being a part of an ethical organization [72]. Being the workers of a socially responsible organization, employees show a greater level of intrinsic motivation due to their organization’s moral norms and values. In addition, past literature on CSR suggests that employees feel pride, respect, and trust in their organization due to its ethical commitment, which ultimately improves their mental health and wellbeing [73]. Past literature indicates that intrinsic motivation could be a significant mediator in reducing the turnover intentions of employees [74,75]. Moreover, with respect to self-concept theory, employees of a socially responsible organization have the belief that there is value congruence between them and their employer as their self-concept, organizational values, and mission have many things in common. This value congruence is likely to reduce a value conflict in employee–employer relationships, ultimately improving employees’ intentions to stay with an organization. Please refer to Figure 1 for research model. Therefore:

**H4.**  *ERCER policies of an organization positively relate to the intrinsic motivation of employees.*

**H5.**  *There is a mediating role of intrinsic motivation between ERCSR and employees’ turnover intentions.*

## 3. Methodology 

### 3.1. Unit of Analysis, Sample, and Procedure 

As specified at the onset of this study, the hospitality sector of Pakistan was the target segment of this study. To represent the hospitality sector in the country, we selected upscale hotels. The reason to include upscale hotels in the data collection process is that all upscale hotels in the country carry out different designated CSR programs and communicate to the internal and external audience how they are fulfilling their social responsibilities for the larger benefits of society and the community. Usually, such CSR-related information is available on the web pages of different upscale hotels (for example, Serena, Avari, Marriot, Carlton, Regent, Pearl Continental, Ramada Plaza international, etc.). From an economic perspective, almost 7% contribution to Pakistan’s GDP is associated with the hospitality sector [76]. Mega-cities of Pakistan, like Karachi, Lahore, Faisalabad, and Islamabad, are famous for hotel operations. Especially, Lahore and Islamabad have special room for this sector because both these cities have many attractions for national and international tourists. This is one of the reasons that almost all national and international hotel chains in the country exist in these cities. Especially Lahore and Islamabad are the most visited cities by tourists in Pakistan [77]. Because both cities are famous for tourism and hospitality, and most of the hotel chains (national and international) exist in Lahore and Islamabad, this study considered these cities as the sampled ones to represent the hospitality sector. 

For this study, we selected Lahore and Islamabad as the base cities for the data collection. In this respect, we communicated our research objectives to the management of different upscale hotels with a request to allow us to have direct facial contact with the employees. Some of the hotels responded positively to our invitation, which we really acknowledge. To proceed further, we devised a proposed scheduled plan to visit different hotels for the data collection. Particularly, the data collection activity was completed in around a two months’ time frame (January to March 2022). The employees (managers and non-managers) of different upscale hotels were the respondents of this study, thus, the unit of analysis was “individual employees.” Please refer Table A1 for data.

### 3.2. Instrument 

A self-administered questionnaire was designed on a five-point Likert scale for this study. The items measuring different variables were adapted from different published sources. In addition, the questionnaire was assessed by field experts for its suitability and accuracy [78,79]. The finalized form of the questionnaire consists of two major parts, among which the first part was related to general socio-demographic information, whereas, in the other part, we collected employees’ responses related to the variables of this study. We applied a multistage data collection strategy (two-wave). This was considered to reduce employee fatigue in filling the responses and to avoid a possible issue of social desirability and common method variance (CMV). On a further note, we fulfilled the major observations of the Helsinki Declaration [80,81,82,83,84] to avoid any ethical concerns in the data collection process. 

### 3.3. Sample Size and Data Cleaning 

An a priori sample size calculator, developed by Fan [85], was used to estimate the minimum sample size for this research study. The specialty of this calculator lies in its ability to propose a sample size with respect to a specific study. This calculator uses some input information to estimate the sample size for a specific study. The early researchers also mentioned the suitability of this calculator, especially for structural equation modeling (SEM) [86,87]. In this respect, there were four unobserved variables (latent) and 30 observed variables (items). When we provided this information to the calculator, along with other inputs, it showed that the minimum sample size for this survey should be 200. To this end, we distributed 400 questionnaires among the employees of different hotels. As with every survey research, we did not receive all of the distributed questionnaires back. Indeed, we were only able to receive 309 questionnaires back from the respondents. After data cleaning (Table 1) and removal of outliers (Table 2), the final dataset of this study constituted 278 valid responses. Additionally, to detect the outliers, we used the Mahalanobis technique in AMOS software. Further, the normality of the data was assured by checking the skewness and kurtosis values. 

The sample of this study included both male and female respondents, however, compared to female respondents, the percentage of male respondents was greater (72%). The experience level of the employees mostly ranged between 1 to 10 years (79%). Likewise, the ages of most respondents were between 18 to 45 years (Table 3).

### 3.4. Measures

This study’s variables were measured using the already available published scales. There were four variables and ERCSR was the predictor variable. This was measured by using a six item scale developed by Turker [88]. Indeed, this is one of the most famous CSR scales which has largely been used by previous scholars. The original scale consists of a total of seventeen items, however, considering the context of this study, we included six employee related CSR items. Employee turnover intention (ETUI) was taken as the criterion variable in this study for which a four item scale developed by Kelloway, et al. [89] was adapted. Some illustrated items from this scale are outlined below. 

First, this study included the variables like quality of work life (QOWL) and intrinsic motivation (INTM) as the mediators of the relationship between ERCSR and ETUI. To measure these mediators, a sixteen item scale developed by Sirgy, et al. [90] was considered to measure QOWL. The genuine scale was designed to measure employees’ perceptions regarding the lower and higher order quality of their work life. 

Last, the variable of INTM was measured by using a five-item scale developed by Tierney, et al. [91], which is also a very famous and reliable scale to measure INTM. The Cronbach alpha (α) values of each variable were assessed to verify the inter-item consistency (reliability) significance. The statistical output revealed significant results in all cases (ERCSR = 0.871, ETUI = 0.853, QOWL = 0.922, and INTM = 0.868).

### 3.5. Common Latent Factor 

Considering the potential risk of CMV (because the data were collected from a single source), we performed a common latent factor (CLF) test in AMOS software. For this purpose, two measurement models were constructed. The first model did not include any CLF (original four factor model), whereas the other model was developed by including a CLF which was intended to produce a direct effect on each observed item of a variable. To arrive at a conclusion regarding a CMV issue, we analyzed both models’ factor loadings (standardized) to see if there was a significant difference (>0.2). The results revealed that the factor loadings differed minutely (<0.2), which implies that both models produced almost the same factor loading. This was an indication that a CMV issue in this study was not a critical matter and did not require any significant corrective measures to proceed with the data analysis. 

## 4. Results

### 4.1. Reliability and Validity 

In the data analysis phase, we verified the convergent validity and reliability of the variables used in this study. To do this, the standardized factor loadings of each item of a variable were taken to calculate the convergent validity. In this regard, through the standardized factor loadings, we were able to calculate the value of average variance extracted for all four variables (ERCSR, ETUI, QOWL, and INTM). Specifically, we employed the following formula to calculate AVE values. It was observed that all AVEs were above 0.5 (which is a normally accepted level), implying that all AVEs were significant. These values, along with other statistics, are reported in Table 4. The AVE values ranged from 0.572–0.617.

(1) $$\mathrm{AVE}=\frac{\sum_{\dot{i}=1}^{k}\lambda_{i}^{2}}{\sum_{\dot{i}=1}^{k}\lambda_{i}^{2}+\sum_{i=1}^{k}.var\left(\varepsilon i\right)}$$

Likewise, to calculate each variable’s composite reliability (CR), we considered the following formula given in Equation (2). The output showed that all values of CR were above the acceptable level of 0.7. This shows that CR was significant in each case.

(2) CR=((∑λi)2)/(∑λi)2+∑var(εi))……….

### 4.2. Model Fitness

To assess a model fit between theory and the data, we constructed three different measurement models in AMOS. These models were then assessed by comparing different model fit indices (NFI, and CFI), chi-square/degree of freedom, and root means square errors of approximation (RMSEA) values. We summarized the results of these three models in Table 5. As per the results of Table 5, one can see that the hypothesized model showed an excellent model fit with the dataset of this study (NFI = 0.956, CFI = 0.952, *χ*^2^*/df* = 2.436, and RMSEA = 0.053). In contrast, a one-factor model (model 1), and a two-factor model (model 2) showed poor model fit values. This indicates that the hypothesized model (four-factor) shows an excellent fit between theory and the data. 

### 4.3. Correlations 

To know the nature of the relationship between different pairs of variables, we evaluated the correlational values (*r*) between different variables. For the convenience of readers, such a comparison is summarized in Table 5, which shows that different variables varied in terms of nature and magnitude with respect to *r* values. For example, the *r* value between the pair ERCSR–ETUI was negative (*r* = −0.528), which shows the negative nature of the relationship. However, when we compared *r* value between the pair ERCSR–INTM, it showed a positive relationship (*r* = 0.419). All in all, the correlations in all cases (positive or negative) were significant and were not critically high (between 0.8 and 1.00), which shows that a multicollinearity issue did not exist in the dataset of this study. Similarly, another kind of validity, which is known as divergent validity, was also tested by taking the square root of every AVE value and then comparing it with the *r* values. While convergent validity was important to see if the items of a variable were converging on to it or not, divergent validity was also important to measure because it shows the items of one variable are dissimilar with respect to another variable. In this respect, the divergent validity results are presented in Table 6 in the diagonal places. For example, the divergent validity value for ERCSR was 0.757, which was greater than all the *r* values in comparison (−0.528, 0.396, and 0.419), showing that divergent validity was significant. A similar kind of observation can be seen in all other cases. 

### 4.4. Hypotheses Evaluation 

The hypothesized model of this study was evaluated with the help of SEM analysis in AMOS. Because the proposed research model of this study included two mediators, we, therefore, selected the bootstrapping option in AMOS by using a larger bootstrapping sample of 5000. The results of SEM analysis revealed that ERCSR negatively predicted ETUI (0.496(0.052)) which is in line with the theoretical statement of H1. However, in other cases ERCSR🡪QOWL and ERCSR🡪INTM the results were different because ERCSR positively predicted QOWL (0.388(0.048)) and INTM (0.412(0.037)). These results were significant because the confidence interval (both lower and upper), in any case, did not involve a zero point. Thus, H2 and H4 were also accepted in the light of the statistical evidence. 

The results of mediating effects show that both QOWL and INTM significantly mediate between ERCSR and ETUI (ERCSR🡪QOWL🡪ETUI = −0.209; ERCSR🡪INTM🡪ETUI = −0.138). This indicates that the inclusion of QOWL and INTM between ERCSR and ETUI provide a further explanation of how ERCSR is important in reducing the turnover intentions of employees. Therefore, H3 and H5 were also accepted (Table 7). 

## 5. Discussion

The statistical results of this study confirmed the theoretical statement of the first hypothesis by indicating that ERCSR activities of a hotel organization help reduce the turnover intentions of its employees (ERCSR🡪ETUI = −0.496). Indeed, an ethical organization tends to improve its employees’ mental and physical health by providing them with different benefits under its social responsibility program. For example, an organization under an ERCSR policy provides employees with a flexible and healthy working environment that positively impacts their mental health. In addition, the CSR orientation of an organization infuses the feeling among the employees that the organization is ethical, where all employees will be treated fairly without any prejudice. This feeling of fair treatment also helps the employees to work in an ethical organization without any fear. Past literature also acknowledges that when employees work fearlessly in an ethical organization, they feel less stress which ultimately reduces their turnover intentions [56,92]. Therefore, in line with the previous literature [37,53], this study confirms that ERCSR negatively predicts employee turnover intentions in a hospitality context. Moreover, because the tourism and hospitality sector of Pakistan is a labor intensive segment (around 4 million employees), improving the wellbeing and mental health is a matter of public health. To this end, well-planned CSR strategies can improve the mental health of the employees on the one hand. Such strategies can reduce work stressors, on the other hand, ultimately improving the turnover intentions of employees. Hence, the statement of our first hypothesis was empirically confirmed in this study. 

Another important finding of this study was to confirm the mediating effect of quality of work life between ERCSR and employee turnover intentions (ERCSR🡪QOWL🡪ETUI = −0.209). A hotel organization that takes into consideration the welfare aspect of employees is expected to raise employees’ perceptions about the quality of their work life. Thus, such organizations foster the motivation and commitment level of employees through different employee benefit programs under ERCSR policies. Indeed, an organization’s quality of work-life program intends to enhance the satisfaction and mental health of employees. The above finding supports past literature in which it was mentioned that employees’ perception regarding the quality of their work life significantly improves as an antecedent of a firm’s CSR strategies [61,62] which then better explains the negative association between ERCSR and employee turnover intentions by producing a mediating effect in this relationship. More specifically, the prime focus of an ethical hotel organization is to emphasize on the wellbeing of its employees. When employees see such extended efforts of their employer for their wellbeing, it improves their perception regarding the quality of work life at the workplace. This improved level of fulfillment regarding the quality of life, as an outcome of ERCSR, will then correspond positively, and thus the mental health of employees improves. Consequently, employees with an enhanced level of mental health develop more positive feelings about quality of work life, which then motivates them to stay with an ethical organization as long as possible, reducing their turnover intentions. 

Lastly, this study also confirms the mediating effect of intrinsic motivation between ERCSR and employee turnover intentions (ERCSR🡪INTM🡪ETUI = −0.138). In this respect, an employee with a higher level of intrinsic motivation shows extra commitment to complete a task for his or her inner satisfaction, not for external rewards. This further explains the possible negative link between ERCSR and employee turnover intentions in the light of self-concept theory. As the self-concept of intrinsically motivated employees relates to their inner satisfaction, the same is the case for an organization with a high CSR orientation (working for the welfare of all). This reduces a value conflict between employees and the organization, which converts this relationship into a meaningful employee–employer relationship. This is in line with the past literature on positive employee psychology, which shows that employees’ intrinsic motivation improves as their CSR perceptions of their organization improve [70,71]. Being the workers of a socially responsible organization, employees show a greater level of intrinsic motivation due to the moral norms and values of their organization. In addition, employees feel pride, respect, and trust in their organization due to its ethical commitment which ultimately improves their mental health and wellbeing [73] and reduces their turnover intentions.

### 5.1. Theoretical Contributions

This study significantly improves the debate on employee turnover intentions by advancing the existing literature in the following ways. In the first place, the literature on ERCSR is advanced from the perspective of employee turnover intentions. In this respect, as it was specified at the onset of this study that although the literature on ERCSR acknowledges its role in influencing employee behavior, most of such literature investigated the potential role of ERCSR from a positive behavioral aspect of employees [16,17,18,19]. Nonetheless, studies on the relationship between ERCSR and employee turnover intentions are sparse, especially in a hospitality context. Therefore, this study attempts to bridge this knowledge gap by proposing that ERCSR can negatively predict employee turnover intentions in a hospitality context. In the second place, this study is one of the limited studies in the domain of employee behavior, especially employee turnover intentions, that attempts to explain the negative association between organizational factors (for example, ERCSR) and employee turnover intentions by testing the simultaneous effect of a psychological mediator (quality of work life) and personality characteristic (intrinsic motivation), in a unified model. Although the mediating effect of intrinsic motivation and quality of work life was stated in prior literature, however, to the best of our knowledge, no previous study investigated the above relationship in a unified model from a hospitality context where turnover is a critical issue. Specifically, this study extends the theoretical framework developed by Kim, et al. [65]. The authors did a decent job by highlighting the mediating role of quality of work life between CSR and employee turnover intentions, however, they still did not consider the potential role of personality characteristics like intrinsic motivation. Finally, this study contributes to the current literature on hospitality management from the perspective of a developing economy. In this respect, much of the previous literature was carried out in developed or high-income countries. Although the phenomenon of increasing employee turnover is a challenge for the overall global hospitality industry, however, compared to developed countries, hotel enterprises in developing countries do not have abundant resources. Therefore, reducing employee turnover intentions in this sector was important because a high turnover rate has economic consequences for a hotel enterprise. 

### 5.2. Practical Contributions

Equally important to mention is that some practical implications are also offered here for the hospitality sector of Pakistan. In this respect, turnover is one of the biggest evils that exist in this sector. Moreover, the huge economic cost of employee turnover also makes it challenging for a hotel organization to survive and surpass the competitor in the face of competition. Therefore, addressing the issue of employee turnover in this sector is of seminal importance. In this respect, this study proposes ERCSR as a potential motivator that reduces employee turnover intentions significantly. A hotel organization needs to closely monitor its CSR strategies from an employee perspective so that as a result of employee-focused CSR activities, the hotel organization is able to improve the mental health of its workforce, which is very important in reducing employees’ likelihood of leaving an organization in the near future. 

Another important contribution here to the field is the realization of organizational disaster related to employee turnover. Not only a hotel organization has to bear the economic loss as an outcome of employee turnover, but the other forms of organizational disruptions related to employee turnover are also unbearable. For example, when an employee leaves an organization, the knowledge inventory of an organization is also lost. Moreover, the social fabric of an organization is also disrupted. In this respect, a carefully planned CSR program can do wonders for a hotel organization because such programs tend to improve the mental health of employees on the one hand, while the ethical commitment of an organization also creates an emotional bond between the employee–employer relationships. The emotional bond as an outcome of CSR also motivates employees to stay with an organization as long as possible. Hence the turnover intention of employees is reduced due to the CSR activities of a hotel organization. 

Lastly, besides the economic cost of turnover in the hospitality sector, a high employee turnover makes it very difficult for hotel management to satisfy their consumers by providing them with continuous service quality. This is because, in a customer-facing service sector, customers’ interaction and experience with a previous employee give no alternative. When customers see employees in a hotel moving all the time, they are less likely to continue repeat purchases from such a hotel organization. Therefore, an increasing employee turnover rate has a special consequence for this sector because employees are the main source that drives organizational success for a hotel. Thus, to answer all such situations, a hotel organization needs to realize the potential role of ERCSR in mitigating the turnover intentions of its employees. 

### 5.3. Limitations and Possible Future Directions

This study faces some potential issues which may be realized as limitations. The first limitation of this study rests with the geographical consideration as the current study collected the data only from Lahore and Islamabad. Although considering the large number of hotels that operate in these cities, that it was worth collecting the data from hotel employees in these cities, we still suggest including more cities in order to have a better generalizability claim of this research because limiting this study to the extent of two cities, may have limited its potential to reflect the true picture of the whole industry. Another potential issue rests with the nature of the data. The current survey was conducted by following a cross-sectional design in which the information was only collected at a specific time. Although cross-sectional surveys are very common in behavioral studies, establishing causal relationships under a cross-sectional survey method is difficult. Therefore, we suggest employing a longitudinal data design in future studies. Finally, a non-probability sampling method was another potential limitation of this survey. Given that due to different policy and safety issues, most hotels did not agree to share any list of employees with us, which could have served as a sampling frame to apply a probability sampling, we were unable to introduce any probability sampling technique. There is no doubt that probability sampling is regarded as superior compared to non-probability sampling. Therefore, if possible, we suggest in future studies to subscribe to any probability sampling (for example, random sampling) method.

## 6. Conclusions

Although the phenomenon of employee turnover has long existed in organizational science literature, in the recent past, the organizational interest in reducing employee turnover intentions has increased. This is because employee turnover has various organizational disruptions, which include but are not limited to, economic loss, bad reputation, loss of knowledge-skill inventory, etc. Shockingly, the turnover rate in the hospitality sector worldwide has been increasing, which is a critical challenge for the management in this sector. In order to address the issue of employee turnover in this sector, we highlighted the role of ERCSR activities in a hotel organization. Given that the benefits of the social responsibility engagement of an organization were mentioned in the previous literature at different levels, the hospitality sector can reduce employees’ turnover intention by carefully planning a CSR program. For this, we suggest the management of a hotel continuously pay attention to CSR activities, especially related to employees. Similarly, another important conclusion that this study highlights is the role of psychological and personality factors like quality of work life and employees’ intrinsic motivation in reducing turnover intentions. Given that these factors significantly buffer the association between ERCSR and employee turnover intentions, we suggest that a hotel organization designs special training programs that improve employees’ intrinsic motivation and their improved perception of quality of work life. Further, such training programs need to be closely coordinated with the ERCSR activities of an organization so that employees clearly realize their hotel’s ethical orientation, which will ultimately improve their intentions to stay with a hotel for as long as possible.

## Figures and Tables

**Table 1:** Data cleaning, outliers, and response rate.

	Distributed	Returned	Unreturned	Unusable	Outliers	Final
	400	309	134	31	09	278
Percentage	-	77.25	22.75	10.03	29.03	69.50

**Table 2:** Observations identified as outliers.

Observation Number	Mahalanobis d-Squared	p1	p2
285	14.391	0.002	0.037
241	14.122	0.003	0.010
277	10.614	0.014	0.131
216	10.236	0.017	0.132
184	10.129	0.018	0.084
233	9.766	0.021	0.096
36	9.508	0.023	0.094
119	9.034	0.029	0.055
47	8.255	0.041	0.253

**Table 3:** Respondents’ profile.

Demographic	Frequency	%
Gender		
Male	199	71.58
Female	229	28.42
Age		
18–25	33	11.87
26–30	47	16.91
31–35	63	22.66
36–40	49	17.62
41–45	32	11.51
Above 45	54	19.42
Experience		
1–3	52	18.70
4–6	91	32.73
7–10	76	27.34
Above 10	59	21.22
Total	278	100

**Table 4:** Validity and reliability.

Variable	*λ*	*λ* ^2^	E-Variance
ERCSR AVE = 0.572 CR = 0.889	0.720	0.518	0.482
	0.716	0.513	0.487
	0.788	0.621	0.379
	0.811	0.658	0.342
	0.774	0.599	0.401
	0.725	0.526	0.474
ETUI AVE = 0.617 CR = 0.865	0.764	0.584	0.416
	0.810	0.656	0.344
	0.859	0.738	0.262
	0.700	0.490	0.510
QOWL AVE = 0.587 CR = 0.958	0.706	0.498	0.502
	0.733	0.537	0.463
	0.714	0.510	0.490
	0.715	0.511	0.489
	0.802	0.643	0.357
	0.783	0.613	0.387
	0.762	0.581	0.419
	0.819	0.671	0.329
	0.749	0.561	0.439
	0.811	0.658	0.342
	0.744	0.554	0.446
	0.701	0.491	0.509
	0.807	0.651	0.349
	0.868	0.753	0.247
	0.717	0.514	0.486
	0.804	0.646	0.354
INTM AVE = 0.596 CR = 0.880	0.700	0.490	0.510
	0.752	0.566	0.434
	0.828	0.686	0.314
	0.766	0.587	0.413
	0.809	0.654	0.346

Notes: *λ* = Item loadings, CR = composite reliability, ∑*λ*^2^ = sum of square of item loadings, E-Variance = error variance.

**Table 5:** Model fit comparison, alternate vs. hypothesized models.

Model	Composition	*χ*^2^/*df* (<3)	Δ*χ*^2^*/df* -	NFI (>0.9)	CFI (>0.9)	RMSEA (<0.08)
1	1-factor ERCSR + ETUI + QOWL + INTM	7.226	0.697	0.438	0.422	0.239
2	(2-factor) ERCSR + EUTI, INTM + QOWL	6.529	4.093	0.582	0.610	0.178
3	(hypothesized) ERCSR, ETUI, QOWL, INTM	2.436	-	0.956	0.952	0.053

**Table 6:** Correlations and discriminant validity.

Construct	ERCSR	ETUI	QOWL	INTM	Mean	SD
ERCSR	0.757	−0.528	0.396	0.419	2.863	0.712
ETUI		0.785	−0.348	−0.402	2.960	0.693
QOWL			0.766	0.478	3.223	0.588
INTM				0.772	3.082	0.649

Notes: SD = standard deviation, diagonal = discriminant validity values, *p <* 0.001.

**Table 7:** Hypotheses testing.

Hypotheses	Estimates (SE)	*t/z*	*p*-Value	CI
(ERCSR🡪ETUI) (ERCSR🡪QOWL) (ERCSR🡪INTM)	−0.496 (0.052) 0.388 (0.048) 0.412 (0.037)	−09.538 08.083 11.135	0.006 0.000 0.004	−0.633, −0.428 0.467, 0.283 0.592, 0.326
Mediating effects (ERCSR🡪QOWL🡪ETUI)	−0.209 (0.027)	−07.741	0.000	−0.388, −0.127
(ERCSR🡪INTM🡪ETUI)	−0.138 (0.022)	−06.272	0.000	−0.199, −0.110

Notes: CI = 95% confidence interval with lower and upper limits.

## Data Availability

Data will be provided on demand.

## Appendix A

**Table A1:** The Items Used in This Survey.

**ERCSR**
Our company encourages its employees to participate in the voluntary activities
Our company policies encourage the employees to develop their skills and careers
The company of Our hospital primarily concerns employees’ needs and wants
Our company implements flexible policies to provide a good work and life balance for its employees
Our company’s decisions related to the employees are usually fair
Our company supports employees who want to acquire additional education
**ETUI**
I am thinking about leaving this company
I am planning to look for a new job
I intend to ask people about new job opportunities
I don’t plan to be in this organization much longer
**QOWL**
I feel physically safe at work
My job provides good health benefits
I do my best to stay healthy and fit
I am satisfied with what I’m getting paid for my work
I feel that my job at (name of the organization) is secure for life
My job does well for my family
I have good friends at work
I have enough time away from work to enjoy other things in life
I feel appreciated at work at (name of the organization)
People at (name of the organization) and/or within my profession respect me as a professional and an expert in my field of work
I feel that my job allows me to realize my full potential
I feel that I realize my potential as an expert in my line of work
I feel that I’m always learning new things that help do my job better
This job allows me to sharpen my professional skills
There is a lot of creativity involved in my job
My job helps me develop my creativity outside of work
**INTM**
I enjoy finding solutions to complex problems
I enjoy coming up with new ideas for products
I enjoy engaging in analytical thinking
I enjoy creating new procedures for work tasks
I enjoy improving existing processes or products
